# HEALTH CARE EMPLOYERS’ PERSPECTIVES ON SKILLS REQUIRED FOR STUDENT SUCCESS IN THE WORKPLACE

**DOI:** 10.1093/geroni/igad104.1689

**Published:** 2023-12-21

**Authors:** Phyllis Cummins, Runcie Chidebe, Rita Karam, Jenna Kramer

**Affiliations:** Miami University, Oxford, Ohio, United States; Miami University, Oxford, Ohio, United States; RAND Corporation, Santa Monica, California, United States; RAND Corporation, Arlington, Virginia, United States

## Abstract

Community colleges (CCs) play a critical role in educating healthcare workers that are important to an aging population. This study investigated the role literacy, numeracy, and problem-solving skills play in CC student success in the classroom and the workplace. Semi-structured virtual and in-person interviews were completed with CC administrators (n=9) and faculty (n=6), along with employers (n=10) who hire students from CCs. We focus on healthcare employers (i.e., home healthcare, nursing homes and hospitals) and the skills they value along with skills that are lacking in CC hires. Examples of health care occupations filled by CC students include certified nursing assistants, qualified medication aides, licensed practical nurses (LPNs), and registered nurses (RNs). Employers reported working closely with CCs, including providing opportunities for clinical rotations, serving on program advisory councils, and providing input into program development. Numeracy skills, such as measuring medication dosages, critical thinking skills, and problem-solving skills, including conflict resolution, are important and expected by employers. Communication skills are also highly valued, especially the ability to communicate on the level of the nursing home resident or hospital patient, along with their family members. Employers noted that LPNs and RNs sometimes have limited leadership and supervisory skills and remarked they are working with CC faculty and administrators to identify mechanisms (e.g., role playing, case studies) to improve those skills. Findings from this project will inform both CC administrators and employers regarding skills that are necessary to succeed as students and healthcare workers to meet the needs of an aging population.

